# Effects of 1,8-cineole on neuropathic pain mediated by P2X2 receptor in the spinal cord dorsal horn

**DOI:** 10.1038/s41598-019-44282-4

**Published:** 2019-05-27

**Authors:** Xiao-bo Zheng, Ya-ling Zhang, Qing Li, Yi-guo Liu, Xiang-dong Wang, Bao-lin Yang, Gao-chun Zhu, Cong-fa Zhou, Yun Gao, Zeng-xu Liu

**Affiliations:** 1 Department of Anatomy, Medical School of Nanchang University, Nanchang, 330006 Jiangxi People’s Republic of China; 2 Jiangxi Health Vocational College, Nanchang, 330052 Jiangxi People’s Republic of China; 3 Grade 2018, Medical School of Tongji University, Shanghai, 310000 People’s Republic of China; 4 Department of physiology, Basic Medical School, Nanchang University, Nanchang, Jiangxi 330006 People’s Republic of China

**Keywords:** Spinal cord injury, Neuronal development

## Abstract

As an intractable health threat, neuropathic pain is now a key problem in clinical therapy, which can be caused by lesions affecting the peripheral nervous systems. 1,8-cineole is a natural monoterpene cyclic ether present in eucalyptus and has been reported to exhibit anti-inflammatory and antioxidant effects. Research has shown that 1,8-cineole inhibits P2X3 receptor-mediated neuropathic pains in dorsal root ganglion. The P2X2 and P2X3 receptors participate in the transmission of algesia and nociception information by primary sensory neurons. In the present study, We thus investigated in the spinal cord dorsal horn whether 1,8-cineole inhibits the expression of P2X2 receptor-mediated neuropathic pain. This study used rats in five random groups: group of chronic constriction injury(CCI) with dimethysulfoxide control (CCI + DMSO); group of CCI; sham group(Sham); group of CCI treated with a low dose 1,8-cineole (CCI + 50 mg/kg); group of CCI with a high dose (CCI + 100 mg/kg). We observed the effects of 1,8-cineole on thermal withdrawal latency (TWL) and mechanical withdrawal threshold (MWT). We examined P2X2 receptors mRNA change in rat spinal cord dorsal horn by *In situ* nucleic acid hybridization(ISH) and Quantitative realtime polymerase chain reaction (qRT-PCR) methods. Western Blotting and Immunohistochemical staining methods were used to observe P2X2 receptor protein expressions in the rat spinal cord dorsal horn. It demonstrated that oral administration of 1,8-cineole inhibits over-expression of P2X2 receptor protein and mRNA in the spinal cord and dorsal horn in the CCI rats. And the study explored new methods for the prevention and treatment of neuropathic pain.

## Introduction

It is known that Neuropathic pain can arise from lesions which affect the central or peripheral nervous systems. At present, the most important clinical treatment for neuropathic pain is chemical drug therapy. However, due to the complex mechanism of neuropathic pain, there is still no drug that is effective for all neuropathic pain diseases. Now the commonly used therapeutic drugs include anti-epileptic drugs, antidepressants, NMDA antagonists, and opioid analgesics, etc^1,2^. However, their long-term use has side effects that cannot be ignored. For example, anti-epileptic drug gabapentin is currently used in the treatment of neuropathic pain, producing a good effect on the central and peripheral neuropathic pain, but it is easy to cause dizziness, lethargy, and peripheral edema, wherefore its long-term use can cause the risk of movement disorders and secondary infections. Therefore, based on new molecular targets, finding an analgesic that is powerful, safe, effective and low tolerant has become a hot topic in pain treatment research.

1,8-cineole (1,3,3-trimethyl-2-oxabicyclo[2.2.2]octane, also known as eucalyptol), is a monoterpene present in many plant essential oils such as from rosemary and eucalyptus^3^. It exhibits anti-inflammatory and antioxidant effects according to reports^4–6^. This material was used to treat sinusitis, chronic rhinitis and bronchitis as well as asthma^7^. It could reduce the spread of infectious bacteria according to some studies^8,9^. It inhibits prostaglandin and cytokine by stimulating monocytes *in vitro*^10^ and also possesses good transdermal permeation for many drugs^11,12^. Patch clamp techniques were used in our previous studies^13^, which showed that cineole can activate TRPA1 channels of dorsal root ganglia (DRG) and consequently, in the substantia gelatinosa neurons, it can increase the spontaneous excitatory postsynaptic currents. This suggests that 1,8-cineole is a potential candidate for neurodegenerative diseases.

Currently, most studies usually consider these ion channels: channel of acid-sensing ion, channel of TRP and channel of ATP-gated ion. For extracellular ATP, in the nerve systems, it is generally regarded as either a crucial co-neurotransmitter or sole neurotransmitter in most nerves^14,15^. It is also known that the P2 family of receptors covers G protein-coupled receptor (P2Y receptor) with more diverse agonist profiles and ATP-gated ion channel (P2X purinergic receptor), which function as a media for nucleotides signalling^16,17^. The P2X receptor opens responding to ATP binding as a nonselective cation channel, which allows for rapid ion flows (Ca^2+^, Na^+^, K^+^) across the membrane. The extracellular ATP interacts with P2X receptors and consequently stimulates the sensory neurons^18–21^. P2X2/3 subtypes use primary sensory neurons while transmitting the nociception and algesia information^22,23^. The selective P2X2/3 and P2X3 receptor antagonists, in experimental pain models, effectively reduce neuropathic pain^24–26^. The CCI rats behave analogously with the neuropathic pain conditions of human beings, which were therefore used as the neuropathic pain model^27^.

Our previous studies demonstrated that, for CCI rats, in the L4-5 DRG, the down-regulation of P2X3 receptor expressions are decreased by 1,8-cineole treatment^28^. In the spinal cord, P2X2 receptor expression was highest in the dorsal horn, with significant neuronal labeling in the ventral horn and intermediolateral cell column^29^. However, in the spinal cord dorsal horn, it is not clear whether 1,8-cineole could inhibit the expression of P2X2 receptor-mediated neuropathic pain. Therefore, in the current work, that question was investigated regarding the effects of oral administration of 1,8-cineole.

## Results

### Behavioural assessment of mechanical withdrawal threshold and thermal withdrawal latency

**Figure 1:**
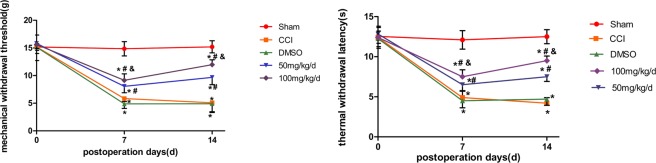
Mechanical withdrawal threshold and thermal withdrawal latency in CCI rats treated with 1,8-cineole. In the CCI + DMSO and CCI groups, the TWL and MWT were decreased than the Sham group; compared to the group of CCI, the two groups of CCI + 100 mg/kg and CCI + 50 mg/kg demonstrated less decreased mechanical and thermal values. The group of CCI + 100 mg/kg had a more pronounced analgesic effect by 1,8-cineole compared with its 50 mg/kg counterpart. ^&^*P* < 0.05 vs. CCI + 50 mg/kg group, ^#^*P* < 0.05 vs. CCI group, *P < 0.05 vs. Sham group. All compared to the corresponding time point (least significant difference test and one-way analysis of variance). n = 6 rats in each group, mean ± SD.

In the CCI group, MWT and TWL became lower than those in the group of Sham (P < 0.05) seven days after the operation and then remained until day 14. In the group of CCI and group of CCI + DMSO, MWT and TWL were not statistically different (P > 0.05). The groups of CCI with 1,8-cineole treatment demonstrated higher MWT and TWL than the group of CCI at 7 and 14 days (p < 0.05). The group of CCI + 50 mg/kg, compared with its 100 mg/kg counterpart, exhibited an increase (P < 0.05) (Fig. 1).

### Effects of 1,8-cineole on P2X2 mRNA expression in spinal cord dorsal horn of CCI rats

**Figure 2:**
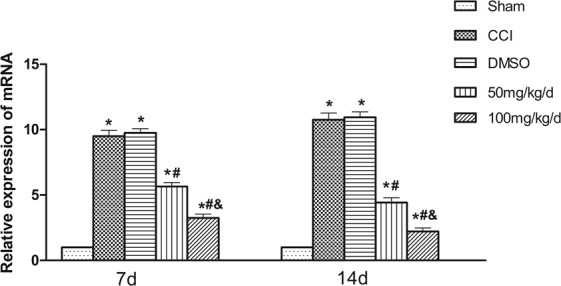
Expression of P2X2 receptor mRNA in spinal cord dorsal horn of 1,8-cineole-treated CCI rats. Relative P2X2 receptor mRNA expression 7 and 14 days following the surgery. In the groups of CCI + DMSO and CCI, the expression was increased than the Sham group; in the two groups of CCI + 100 mg/kg and CCI + 50 mg/kg, the expression was less pronounced. ^&^*P* < 0.05 vs. CCI + 50 mg/kg group, ^#^*P* < 0.05 vs. CCI group, *P < 0.05 vs. Sham group. All compared to the corresponding time point (least significant difference test and one-way analysis of variance). n = 6 rats in each group, mean ± SD.

The groups of CCI and CCI + DMSO demonstrated higher P2X2 receptor mRNA expression than the Sham group (*P* < 0.05); while the former two groups were not different statistically (*P* > 0.05). The groups of CCI with 1,8-cineole treatment showed lower P2X2 mRNA expression levels than the group of CCI (*P* < 0.05). Additionally, compared to the group of CCI + 50 mg/kg, its 100 mg/kg counterpart had lower expression levels of P2X2 mRNA (*P* < 0.05) (Fig. 2).

### *In situ* hybridization results

**Figure 3:**
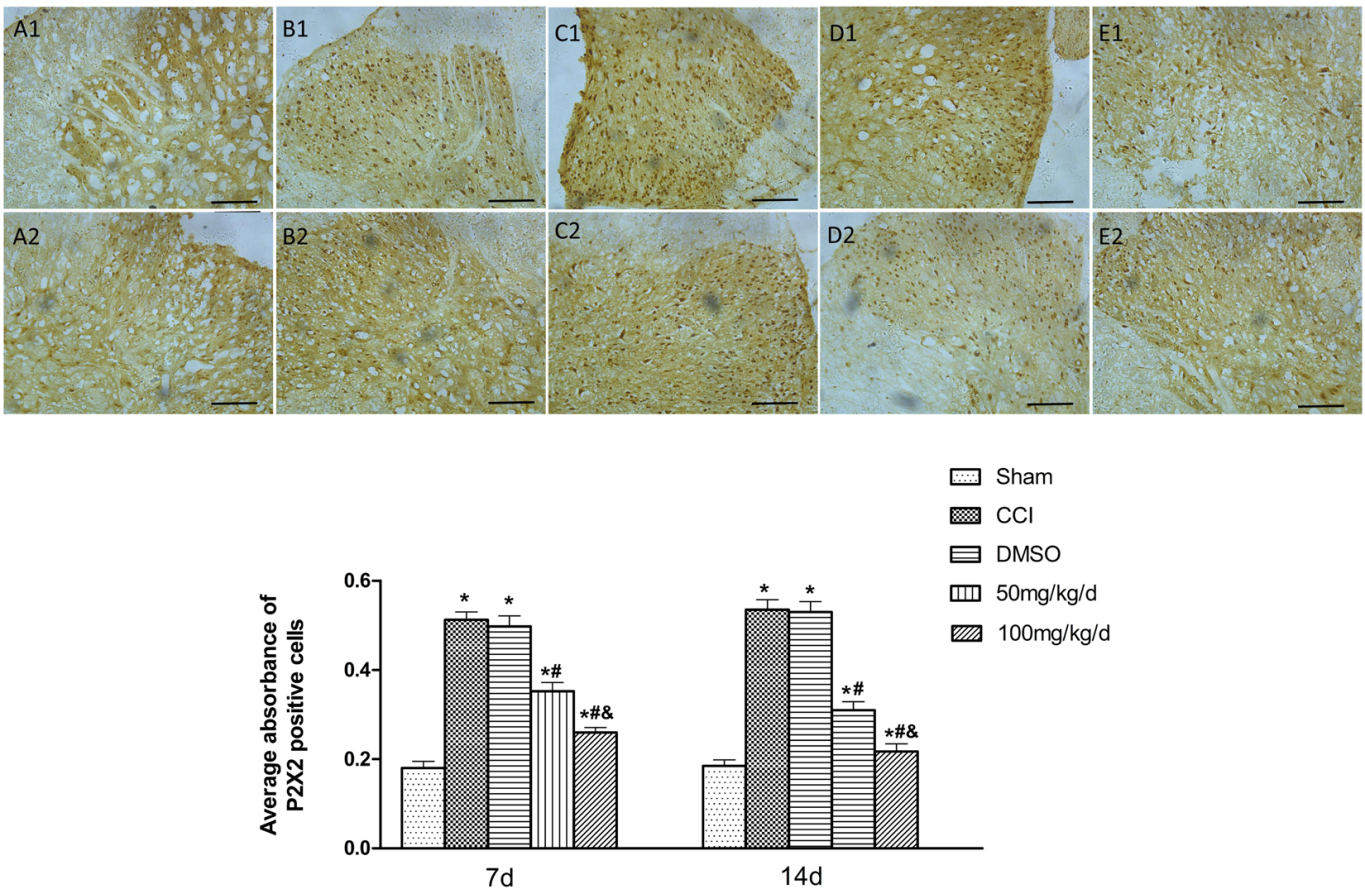
P2X2 mRNA expression in spinal cord dorsal horn. 1 and 2 respectively corresponds to 7 and 14 days after the surgery. (**A**) to (**E**) respectively represents the group of the Sham, CCI, CCI + DMSO, CCI + 50 mg/kg, and CCI + 100 mg/kg. In the two groups of CCI + 100 mg/kg and CCI + 50 mg/kg, P2X2 mRNA showed less upregulation; in the CCI + DMSO and CCI groups, an increased P2X2 mRNA expression was showed compared to the Sham group. ^&^*P* < 0.05 vs. CCI + 50 mg/kg group, ^#^*P* < 0.05 vs. CCI group, *P < 0.05 vs. Sham group. All compared to the corresponding time point (least significant difference test and one-way analysis of variance). n = 6 rats in each group, mean ± SD.

The P2X2 receptor mRNA expression was not changed in the Sham group at 7 and 14 days after the surgery; in the group of CCI, however, the expression was increased significantly comparatively (*P* < 0.05). For the two groups of CCI + 100/CCI + 50 mg/kg, significantly lower P2X2 expression demonstrated compared to the CCI group (*P* < 0.05), though the two groups still showed higher expression than the Sham group. Within the two groups of CCI + 100/CCI + 50 mg/kg, the former exhibited a less pronounced increase (*P* < 0.05). However, the P2X2 receptor mRNA expression was not different significantly among the CCI + DMSO and CCI group (*P* > 0.05) (Fig. 3).

### Effect of 1,8-cineole on P2X2 receptor expression in spinal cord dorsal horn of CCI rats

**Figure 4:**
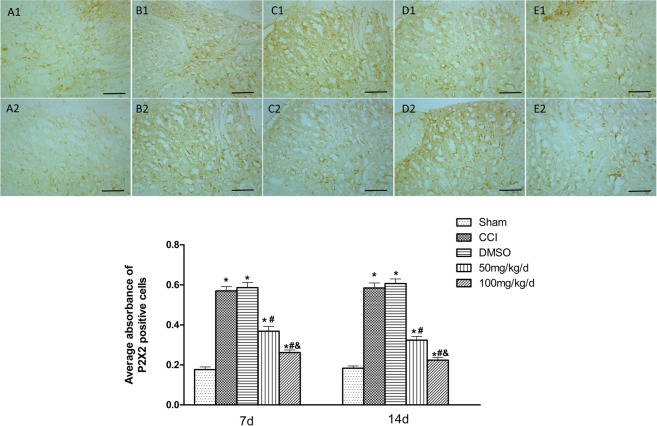
P2X2 immunoreactivity in spinal cord dorsal horn of 1,8-cineole-treated CCI rats. 1 and 2 respectively corresponds to 7 and 14 days after the surgery. (**A**) to (**E**) respectively represents the group of the Sham, CCI, CCI + DMSO, CCI + 50 mg/kg, and CCI + 100 mg/kg. P2X2 receptor immunoreactivity, observed mainly in the cytoplasm 7 and 14 days after the surgery, was shown in yellow-brown. In the group of Sham and the two groups of CCI + 100 mg/kg and CCI + 50 mg/kg, less strong immunore activity was showed, while the groups of CCI and CCI + DMSO were distinctly stained. The mean optical density of P2X2 immunoreactive cells was showed in the following statistical figure. ^&^*P* < 0.05 vs. CCI + 50 mg/kg group, ^#^*P* < 0.05 vs. CCI group, *P < 0.05 vs. Sham group. All compared to the corresponding time point (least significant difference test and one-way analysis of variance). n = 6 rats in each group, mean ± SD, Scale bar: 50 um.

The expression of spinal cord dorsal horn P2X2 receptor was detected by immunohistochemistry. In the groups of CCI + DMSO and CCI, the samples had increased staining intensities for P2X2 receptor than the group of Sham (*P* < 0.05). In the two groups administrated with 1,8-cineole, in comparison with the group of CCI, the ratio of P2X2-positive cells decreased (*P* < 0.05). Within the two groups of CCI + 100/CCI + 50 mg/kg, the former had a significantly decreased ratio of P2X2-immuno labelled cells than the latter (*P* < 0.05) (Fig. 4).

### Effect of 1,8-cineole on P2X2 protein expression in spinal cord dorsal horn of CCI rats

**Figure 5:**
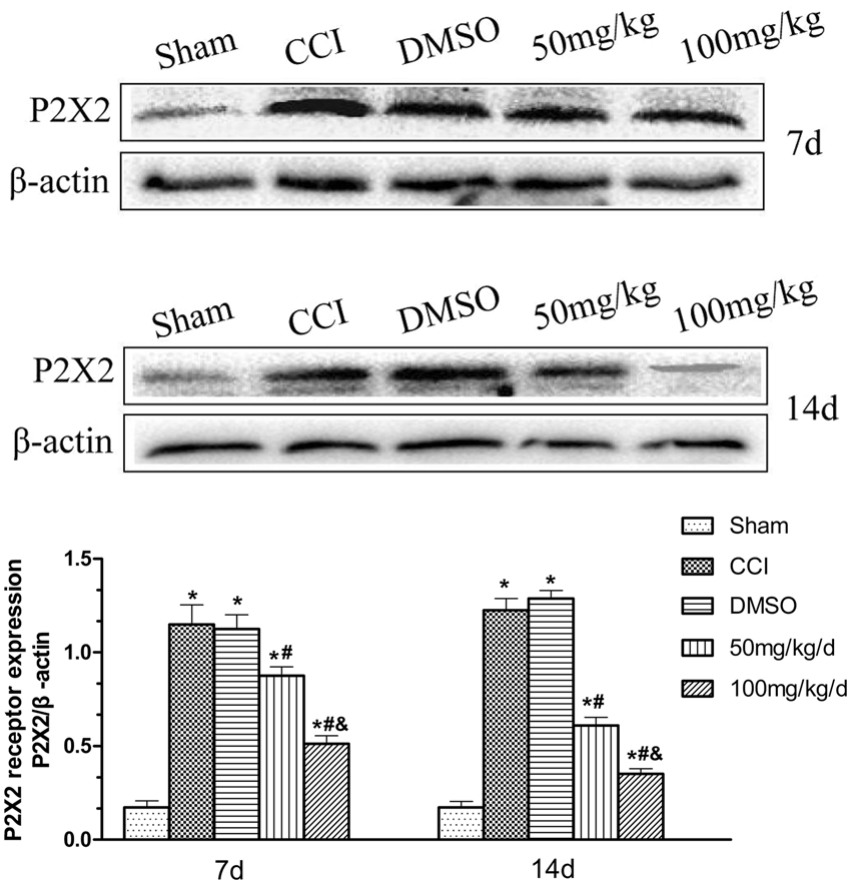
Expression of P2X2 protein in spinal cord dorsal horn of CCI rats treated with 1,8-cineole. In the two groups of CCI + 100 mg/kg and CCI + 50 mg/kg, P2X2 protein showed less upregulation; in the CCI + DMSO and CCI groups, an increased P2X2 protein expression was showed compared with the Sham group. ^&^*P* < 0.05 vs. CCI + 50 mg/kg group, ^#^*P* < 0.05 vs. CCI group, *P < 0.05 vs. Sham group. All compared to the corresponding time point (least significant difference test and one-way analysis of variance). n = 6 rats in each group, mean ± SD.

In this study, western blotting was used to detect the expression of P2X2 protein in spine cord. In comparison with the group of Sham, the CCI group demonstrated significantly enhanced P2X2 protein expression stain values in the spine cord and dorsal horn (*P* < 0.05). But the CCI group and CCI + DMSO group were not statistically different (*P* > 0.05). The 1,8-cineole administrated groups demonstrated lower relative levels of P2X2 protein than the CCI group (*P* < 0.05). Additionally, the group of CCI + 100 mg/kg demonstrated less elevated expression levels of P2X2 protein compared with its 50 mg/kg counterpart (*P* < 0.05) (Fig. 5).

## Discussion

Generally speaking, three orders of neurons are involved in the superfical sensory pathway of trunk and limbs. The first-order neurons with their nuclei in the dorsal root ganglia, carry sensations from the exteroceptors located in the skin of trunks and limbs and enter the posterolateral sulcus of the spinal cord. The axons of second-order neurons in the posterior horns travels up to the ventral posterolateral nucleus (VPL) of the thalamus (the third-order neurons). The trunk and limb receptors sense various external stimuli and convert this stimuli into electrical signals, the primary afferent fibers conduct this electrical signals to the dorsal horn, which is then transmitted by the ascending fibers of the spinal cord to the more advanced nerve center after being transmitted to the dorsal horn by the DRG. The central processes of the horn neurons and the DRG neurons form a primary synapse^30–32^, in which the spinal cord dorsal horn have a role of relaying and processing sensory information. Therefore, for the understanding of the mechanism of neuropathic pain, the spinal and dorsal horn may be a key part.

The CCI model has peripheral and central sensitization characteristics that are very similar to the clinical features of chronic neuropathic pain^33^. This allows for the wide use of this model in neuropathic pain related studies. Its core is to ligature the sciatic nerve with a chrome gut whilst not affecting the blood supply. In our experiments, there was no dyskinesia and autophagy in the hind limbs of the CCI group. In addition, the sensitivity of each CCI rat to mechanical pain and heat sensitive pain was increased at 7 and 14 days after the surgery, i.e. the pain threshold is lowered, indicating that it is a successful model. For neuropathic pain, both cell electrophysiology and immunohistochemistry showed significantly increased P2X2 receptor expression, indicating that the neuropathic pain is closely related to the P2X2 receptor expression^24–26^.

According to the results of immunohistochemistry, *in situ* hybridization, Western blotting or qRT-PCR in this study, each CCI group demonstrated significantly increased expressions of P2X2 receptor mRNA and protein. The groups of CCI with 1,8-cineole administration showed a significant decrease (P < 0.05) than the group of CCI and the group of CCI + DMSO. The CCI + 100 mg/kg group showed significantly lower P2X2 receptor expression than the CCI + 50 mg/kg group (P < 0.05), while the CCI group and CCI + DMSO group were basically the same concerning P2X2 receptor and mRNA expression. The reason why the 100 mg/kg dose group achieved better therapeutic results than its 50 mg/kg counterpart is probably because of the first-step elimination effect after the oral administration. We referred to the relevant literature^34^ and took many pre-experiments, in which we found that if the dose of 1,8-cineole given to the CCI rats is too low, the treatment effect is not obvious; while if the dose is too high, repeated vomiting and even death would occur in the rats. Finally, we chose 50 mg/kg and 100 mg/kg as the reference doses in the treatment. However, the optimal therapeutic dose of 1,8-cineole needs to be established by further studies investigating the toxicity and side effects of this drug.

This study indicates that, for rats having neuropathic pain, in their spinal cord dorsal horn, the over-expression of P2X2 receptor protein and mRNA can be reduced by 1,8-cineole. We speculate that the possible mechanism works in these procedures: ① Oral administration of 1,8-cineole works faster and more effectively; ② Around the lesion, a better microenvironment is created by 1,8-cineole owing to its anti-inflammatory and antiseptic effects^6,7^; β 1,8-cineole relieves structural damage to the other cells (such as Schwann cells, etc.) whilst also promoting the restoration of the injured sciatic nerves^28^.

For the treatment of neuropathic pain, 1,8-cineole may become a new analgesic, though further verifications are needed concerning its mechanisms. For example, the route of administration of 1,8-cineole, the optimal dose for therapeutic effects, as well as the drug metabolism rate of 1,8-cineole in the body still need to be further explored. Additionally the influence of P2X2 receptor on neuropathic pain remains unclear, i.e., whether there are other affecting signal channels. With a deeper understanding of 1,8-cineole in the field of analgesia, it is expected that, in the near future, it will become a major drug in the treatment of neuropathic pain.

## Methods

### Statement

(1) All experiments, including methods and operations were approved by the Animal Care and Ethics Committee of the Medical School of Nanchang University, China. (2) All experimental methods were performed in accordance with guidelines and regulations of the Ethics Committee of the Medical School of Nanchang University, China.

### Animals, drugs and drug administration

For the experiment rats, the Jiangxi Province Traditional Chinese Medicine University Laboratory Animal Science Department provided 120 SD rats, male and female, each with a weight between 200–250 g. The animal certificate has the code SCXK2006-0001. The Committee for Ethical Use of Medical School of the Nanchang university, China. approved all the experiments as per relative international codes. On a clean work bench, the DMSO and 1,8-cineole, both at 2% v/v, were prepared. Five random groups of rats: group of CCI + DMSO (CCI, DMSO administrated), group of CCI (CCI, no administration), group of Sham (sciatic nerves isolated, not constricted), group of CCI + 50 mg/kg (CCI, 50 mg/kg/d 1,8-cineole administrated) and group of CCI + 100 mg/kg (CCI, 100 mg/kg/d 1,8-cineole administrated). The intragastrical administration of 1,8-cineole or equal quantity of DMSO was made daily from the first postoperative day. Each of the above groups were subdivided into two groups, with 12 rats per group, respectively for 7 or 14 days treatment and time point.

### Model production

With CCI model established^25^, 1% sodium pentobarbital (40 mg/kg, i.p.) was used to anesthetize the rats. The left sciatic nerve in the rat thigh was ligated 1 mm apart using 4–0 catgut, with an appropriate intensity not affecting the nerve blood supply. This procedure, except the ligation of nerve injury, was applied to the group of Sham.

### Behavioral test

At 7 and 14 days after the surgery, all the groups of rats were placed in a VonFrey pain threshold measuring glass instrument, the bottom of which was made of barbed wire. The device was placed on a horizontal surface, let the rats adapt to this tool for 15–20 minutes at normal temperatures and in a quiet environment. Von Frey filaments and BME-410C Thermal Paw Stimulation System (Aesthesio, Danmic, CA, USA) were used respectively for the tests of TWL and MWT. The experiments were performed three times with the average value to be obtained and investigated^35,36^.

### qRT-PCR

PBS was used to isolate and immediately wash the rat spinal cord dorsal horn lumbosacral enlargement. A FastQuant RT Kit with gDNase (Tiangen Biotech Co., Beijing, China) was used to synthesize cDNA with total RNA of 2 µg. TRNzol Universal Reagent (Beijing Tiangen) was used to prepare the total RNA samples. Primer Express 3.0 (Applied Biosystems, Inc., Foster City, CA, USA) was used to design the primers. The sequences were defined in the following way: P2X2 receptor, forward 5′-GGTGGTAGTGCCGTTTATCT-3′, reverse 5′-AAGGGCGGTGTCATTGGA-3′; β-actin, forward 5′-AAGATCCTGACCGAGCGTGG-3′, reverse 5′-CAGCACTGTGTTGGCATAGAGG-3′. The ΔΔCT method was used to quantify the gene expression, with the threshold cycle denoted by CT. The relative levels of the target genes normalized to those of the samples with the lowest CT were reported as 2−ΔΔCT. A SuperReal PreMix Plus (SYBR Green) was used to perform quantitative PCR in an ABI PRISM® 7500 Sequence Detection System (Applied Bio-systems, Inc.).

### *In situ* nucleic acid hybridization

Firstly 1% sodium pentobarbital (40 mg/kg, i.p.) was used to deeply anaesthetize the rats and then physiological saline, 4% paraformaldehyde and 0.1% diethylpyrocarbonate (DEPC) were perfused through the ascending aorta successively. Afterwards, 4% paraformaldehyde with 0.1% DEPC was used to incubate the tissues overnight at 4 °C. On the next day, sucrose solutions of 15% and 30% at 4 °C were sequentially used for overnight dehydration. A cryostat was used to slice the tissues into sections of 10 µm in thickness before being placed on poly-L-lysine covered glass slides. These sections were then incubated for 10 minutes in 0.4% Triton X-100/PBS after they were washed using 0.01 mol/L PBS for 3 × 5 min. Protease K (5.0 μg/ml) in PBS was then used to incubate the sections at 37 °Cfor 10 minutes. 4% paraformaldehyde was used to fix the sections for 5 minutes to stop the protease activity before being washed off the fixative by 2 × 3 min washes with PBS. Sodium chloride of 0.6 mol/L and sodium citrate of 0.06 mol/L (2 × SSC) were then successively used to wash the sections for totally 10 min. To allow for hybridization, a humid chamber at 37 °C was used to keep the coverslip covered smear for 30 minutes. Pre-hybridization buffer was used to wash the sections for three times before incubating these firstly at 60 °C for 30 minutes and then at 45 °C for two hours, respectively with a hybridization buffer containing 5 pM of each probe and with a humid chamber. Finally wash buffer and rinse buffer were used to respectively twice wash and rinse the smear before it was dried in the darkness.

### Immunohistochemical staining

4% paraformaldehyde was used to perfuse and fix the spine cords of the rats and then post fix these for additional 24 h before being transferred to 15% and 30% sucrose solutions at 4 °C for overnight dehydration. The segments of the spine cord lumbosacral enlargement were longitudinally cut into serial sagittal frozen sections of 10-um in thickness on a cryostat after being embedded in optimal cutting temperature compounds. The frozen slides were washed twice with PBS every five minutes after being dried for 10 minutes at room temperatures in the air. Rabbit anti-P2X2 antibody (1:500; Abcam) was then used to incubate the sections at 4 °C overnight after these were blocked for 15 minutes at room temperatures using 10% normal goat serum (Boster, Wuhan, China). An additional incubation for 90 minutes at 37 °C was then performed using horseradish peroxidase-labelled goat anti-rabbit IgG (1:100; Boster). After that, PBS was used to rinse the slices for three times at 5-minute intervals, which were then dehydrated, permeabilized, and mounted after being developed using 3,3′-diaminobenzidine and stained using hematoxylin. From each rat, three sections were obtained, and for each section light microscope (Olympus, Tokyo, Japan) was used to randomly select three non-overlapping fields.

### Western blotting

The spinal cord dorsal horn lumbosacral enlargement was homogenized in RIPA lysis buffer containing a protease inhibitor (100 mg/mL phenylmethylsulfonyl fluoride; 1 mg/mL aprotinin; 0.1% sodium dodecyl sulfate (SDS); 1% Nonidet P-40; 0.02%sodium deoxycholate; 150 mMNaCl; and 50 mM Tris-Cl, pH 8.0). Collect the supernatant after centrifuging the homogenate at 12,000 rpm for 10 min. A 6× loading buffer was used to dilute the supernatant, which was then heated for five minutes at 95 °C. A gel electrophoresis system of Bio-Rad 10% SDS polyacrylamide was used to separate the proteins from the samples that contain the same quantity of protein (30 µg) and to transfer the proteins to polyvinylidene fluoride membranes. 5% non-fat dried milk was used to block the membrane for two hours at room temperatures in 1× Tris-buffered saline containing Tween-20 (TBST). A rabbit Polyclonal anti-P2X2 antibody(1:500; Abcam) and a mouse monoclonal anti-β-actin antibody (1:5,000, ABclonal) were then used to incubate the membrane at 4 °C in blocking buffer overnight. A 1× TBST and a horseradish peroxidase-conjugated secondary antibody (1:5,000, ABclonal) (goat anti-rabbit IgG or goat anti-mouse IgG) were used to wash the membranes for 3 × 10 min and incubate these for one hour at room temperatures respectively in blocking buffer. After a second time washing, a Bio-Rad system and enhanced chemiluminescence were then used to visualize the labeled proteins. To quantify the band intensity, Image-Pro Plus software was adopted. The relative band intensities of the target proteins were normalized to those of the respective β-actin internal controls^37^.

### Statistical analysis

The software of SPSS 19.0 (SPSS, Chicago, IL, USA) was used to analyze the data, which are expressed as the mean ± SD. One-way analysis of variance ANOVA was used to explore the intergroup differences and the least significant difference as a post hoc test, in which P < 0.05 was regarded as significant statistically.

## Data Availability

All data generated or analysed during this study are included in this published article.
